# 

**Published:** 1895-11

**Authors:** 


### EDITORIAL.

### Ohio State Dental Society.

The annual meeting of this body will be held in Columbus, on the first Tuesday, the 3rd of December next. The indications are that this will be one of the best meetings of this society ever held, both in attendance and in the interest that will attend the occasion. There is quite a number of vital questions that will, or ought to be considered at that time and there is no doubt but that they will.

A program is being prepared, but is not completed; we are able however to give the following as a part of it, viz:

"Does the Practice of a Specialty of Medicine Tend to Make a Man Narrow-minded?" By Dr. C. M. Wright, of Cincinnati.

"Diagnosis of Heart Lesions, Previous to Administering Anæsthetics." By Dr. W. H. Whitslar, of Cleveland.

"Dental Education." By Dr. J. T. Jackman, of Cleveland.

"The Advent of Electricity." By Dr. Edward C. Hall, of Columbus.

"Selection of Anæsthetics and their Administration." By Dr. A. O. Ross, of Columbus.

"Report of Two Cases of Neuralgia of Second Branch of the Fifth Pair—Operation—Relief." By Dr. W. D. Hamelton, of Columbus.

"Some Thoughts in Connection with the Combination of Amalgam and Gold." By Dr. F. W. Knowlton, of Akron.

"Implantation of a Tubed Tooth." By Dr. J. B. Snyder, of Bryan.

A paper—subject not yet given. By Dr. H. T. Smith, of Cincinnati.

A paper—subject not yet given. By Dr. Grant Mitchell, of Canton.

A paper—subject not yet given. By. Dr. L. L. Barber, of Toledo.

There will be clinics, by Drs. Whitslar, Condit, Snyder and a number of others. Some of these we know from personal observation will be interesting and instructive.

The above constitutes only a minor part of the program, the whole of which we are assured, will be distributed in a few days.

A most cordial invitation is extended to all who possibly can, whether in or out of the State, to be present. If there are any critics of the former work of the society, they above all others, ought to be present and help to put things to rights. If there are any who can afford to stay away it is those who have in the past received the most from these meetings. Those who have not been feeding at this table had better come in and partake.

### Ohio State Dental Society.

The annual meeting of this Society will be held in Columbus on the first Tuesday of December, 1895.

There is a number of important subjects that will require attention at that time; one very important matter is the consideration of the Dental Law of the state. The question: "Is it accomplishing all that was expected when it was enacted;" and if not, what can be done to make it better?

The subject of Association work in Ohio is another that certainly demands serious consideration; other States are outstripping Ohio in this matter, and it certainly ought not to be so; and will not if the State Society does its duty.

The officers having its interests especially in charge should be promptly on the alert; a large and active meeting is at this time all important.

### CONTENTS.

COMMUNICATIONS.

### THE DENTAL REGISTER,

A MONTHLY MAGAZINE DEVOTED TO THE INTERESTS OF THE

### DENTAL PROFESSION.

*Terms Reduced to* $2.00 *Per Year. Single Copies,* 25 *Cents.*

### EDITED BY PROF. J. TAFT, D.D.S.

—AND—

### Published by SAM'L A. CROCKER & CO., Ohio Dental and Surgical Depot.

The volume commences in January and closes in December.

The REGISTER being issued monthly is always fresh, therefore Indispensable to the practitioner who desires to keep posted up with the new inventions and improvements which are daily developed. Neither expense nor effort will be spared to give value and variety to its contents. Articles will be illustrated with well executed engravings whenever they will add to the interest or assist to explanation.

Its extensive circulation and frequency of issue make it one of the BEST DENTAL ADVERTISING MEDIUMS in the United States.

All subscriptions must begin with January or July.

Local or special notices, five lines or less, will be inserted for $3 each insertion; over five lines, 50 cts. per line.

All advertising bills payable in advance, or at furthest, after the issue of the first number containing the advertisement. All transient advertisements to be paid for in advance.

☞CONTRIBUTIONS SOLICITED.

Exclusively Editorial Communications should be addressed to the Editor.

The latest number of the REGISTER may be ordered with or without other goods at any time, and all letters should be addressed to

